# Supplementary material for: Proteomic Profiling of the First Human Dental Pulp Mesenchymal Stem/Stromal Cells from Carbonic Anhydrase II Deficiency Osteopetrosis Patients
Source: Int J Mol Sci. 2020 Dec 31;22(1):380. doi: 10.3390/ijms22010380 (PMC7795265; doi:10.3390/ijms22010380)
Supplement: Supplementary file 1 [file ijms-22-00380-s001.zip › Supplementary Table S4-Antibodies-clones -color and vendor.docx]

**Supplementary Table S4- List of antibodies used in the study (clones, colors, vendor)**

**Description of the Human MSC Analysis Kit by BD Stemflow^TM^**

| **Antibodies** | **Color and Clones** | **Company** |
| --- | --- | --- |
| hMSC Positive Cocktail | CD90 FITC (Clone: 5E10) |  |
| (Cocktail to positively identify hMSCs) | CD105 PerCP-Cy5.5 (Clone: 266) | BD Biosciences |
| CD90+ CD105 PerCP+ CD73 APC | CD73 APC (Clone: AD2) |  |
| Corresponding Isotype Control for hMSC positive  CD34 P+ | mIgG1, κ FITC (Clone: X40)  mIgG1, κ PerCP-Cy5.5(Clone: X40)  mIgG1, κ APC ((Clone: X40) | BD Biosciences |
| PE hMSC Negative Cocktail | CD34 PE (Clone:581) |  |
|  | CD11b PE (Clone: ICRF44 | BD Biosciences |
| (Cocktail to identify potential contaminants) | CD19 PE (Clone: HIB19) |  |
|  | CD45 PE (Clone: HI30) |  |
|  | HLA-DR PE (Clone: G46-6) |  |
| PE hMSC Negative Isotype Control Cocktail | mIgG1, κ PE (Clone: X40) | BD Biosciences |
| Corresponding Isotype control for PE hMSC | mIgG2a, κ PE (Clone:G155-178) |  |
| Negative Cocktail  (Compensation control/MSC positive drop-in) | CD90 FITC (Clone: 5E10) | BD Biosciences |
| PE Mouse Anti-Human CD44  (positive drop-in)  (Compensation control) | CD44 PE  (Clone: G44-26) | BD Biosciences |
| CD105 PerCP-Cy5.5 Mouse Anti-Human CD105  (Compensation control) | CD105 PerCP-Cy5.5 (Clone: 266) | BD Biosciences |
| CD73 APC Mouse Anti-Human CD73  (Compensation control) | CD73 APC (Clone: AD2) | BD Biosciences |
| PE Mouse IgG2b, κ Isotype Control  (Corresponding Isotype Control for PE Mouse Anti-Human CD44, when used as a drop In) | mIgG2b κ (Clone: 27-35) | BD Biosciences |
